# Pay-to-Win Gaming and its Interrelation with Gambling: Findings from a Representative Population Sample

**DOI:** 10.1007/s10899-021-10042-1

**Published:** 2021-06-09

**Authors:** Fred Steinmetz, Ingo Fiedler, Marc von Meduna, Lennart Ante

**Affiliations:** 1 Faculty of Business, Economics and Social Sciences, Universität Hamburg, Von-Melle-Park 5, 20146 Hamburg, Germany; 2 Faculty of Arts and Science, Research Chair On Gambling, Concordia University, 1455 Boulevard de Maisonneuve O, Montréal, QC H3G 1M8 Canada

**Keywords:** Pay-to-Win, Online gambling, Problem gaming, Problem gambling, In-game payments, Consumer protection

## Abstract

Pay-to-Win gaming describes a common type of video game design in which players can pay to advance in the game. The frequency and value of payments is unlimited, and payments are linked to players’ competitiveness or progress in the game, which can potentially facilitate problematic behavioral patterns, similar to those known from gambling. Our analyses focus on assessing similarities and differences between Pay-to-Win and different forms of gambling. Based on a survey among 46,136 German adult internet users, this study presents the demographic and socio-economic profile of (1) Pay-to-Win gamers who make purchases in such games, (2) heavy users who conduct daily payments, and (3) gamers who are also gamblers. Motives for making payments were assessed and participation, frequency and spending in gambling by Pay-to-Win gamers are presented. To assess the similarity of Pay-to-Win gaming and gambling, we tested whether Pay-to-Win participation, frequency of payments and problematic gaming behavior are predictors for gambling and cross-tested the opposite effects of gambling on Pay-to-Win. We find that Pay-to-Win gamers are a distinct consumer group with considerable attraction to gambling. High engagement and problematic behavior in one game form affects (over)involvement in the other. Common ground for Pay-to-Win gaming and gambling is the facilitation of recurring payments.

## Introduction

Gaming and gambling have been converging ever since the dawn of the internet (Reynolds, 2016) and distinguishing between gaming and gambling has become increasingly complex (Fiedler et al., 2018; King & Delfabbro, 2020; King et al., 2015). The implementation of gaming-elements in gambling (‘gamification’) and gambling-elements in gaming (‘gamblification’) is used by operators to promote players’ commitment and enhance their engagement (Tone et al., 2014; Zanescu et al., 2020). One example of this convergence is loot boxes, which are items in video games that can be bought by players and contain randomized rewards (DeCamp, 2021; Larche et al., 2019; Zendle et al., 2020a; Von Meduna et al., 2020; Macey & Hamari, 2019; King & Delfabbro, 2018). While loot boxes represent a gamblification of gaming in the sense that they incorporate a gambling-like element through a mechanism of chance, another phenomenon has received comparably less attention from academia: Pay-to-Win gaming.

We define Pay-to-Win games as those video games that offer the possibility to spend money on content, items or events which help a player to actually advance in the game and not for mere cosmetic reasons. While the optional purchase of in-game items, events and content is a common feature in online games, Pay-to-Win products are functional in that they afford players a distinct advantage in the game and increase the likelihood of winning. For example, money can be spent on a powerful sought-after item, to progress to a higher level, or to increase the strength of an avatar. As such, a loot box can be a Pay-to-Win product, if its contents yield functional characteristics, which increase players’ likelihood of winning. Pay-to-Win games have been criticized by developers and gamers for causing frustration and the feeling that the games lack fairness (Alha et al., 2018).

Pay-to-Win ‘gamblifies’ gaming not by introducing elements of chance but by potentially provoking psychological responses similar to gambling. For example, by linking in-game purchases with virtual superiority over other players, Pay-to-Win games intensify competition and monetize individuals’ competitiveness. In gambling, competitiveness leads to higher engagement (Mowen, 2004; Mowen et al., 2009) and presents a risk factor for problem gambling (Parke et al., 2004). In combination with the absence of limits for frequencies and amounts of spending, Pay-to-Win games could promote similar problematic behavioral patterns as those known from gambling. Another important implication of this is the variability of spending per player: Many players do not spend anything, some spend a little, and a small number of players, so called ‘whales’, pay large sums. This leads to a high concentration of revenues generated from a minor fraction of the players–a typical characteristic of gambling (Fiedler et al., 2019). The combination of these coherences indicates a potential relation of Pay-to-Win and gambling, which needs to be addressed by researchers.

Although Pay-to-Win-elements are omnipresent in the gaming industry (Zendle et al., 2020b), the quantity of literature focusing on this phenomenon does not reflect its relevance. This refers to research on Pay-to-Win as a specific form of gaming, for its relation to gambling as well as regulatory implications. The objective of this study is to contribute to the exploration of this phenomenon by investigating the motives and socio-demographics of Pay-to-Win gamers and the relation to gambling. Using a large-scale German panel, we seek to identify socio-demographic and behavioral similarities between Pay-to-Win gamers and gamblers, excessive gamers and problem gamblers, and players who participate in both.

## Literature Review and Research Questions

Research has been accumulating over the last decade on the motives for players to buy in-game content, items or events (Hamari & Keronen, 2016). It can be argued that only the sale of functional in-game benefits makes a Pay-to-Win game, which highlights the importance of distinguishing products of functional and non-functional character. For non-functional in-game items, Marder et al. (2019) consider hedonic and social motives to be dominant. For example, the possession of rare and expensive items, e.g. special skins, can help to establish visual authority or superiority over others. The investment of real money in the game can signal a player’s skill, reputation and dedication. Alha et al. (2018) find that most motives regarding non-functional items also apply to functional items, for example the desire to give something back to the developer or to express appreciation of their work. Nevertheless, the purchase of functional items remains predominately motivated by the desire to advance in the game, even though the Pay-to-Win aspect is widely frowned upon. The question then arises whether within the desire to advance in the game, players’ motives can be further broken down into, for example, the motive to increase the chances of winning, speed up the play, or reduce waiting time. A research gap thus exists not about in-game purchases and products in general but with reference to the motivation of purchasing functional items.

Similar to the motives for buying Pay-to-Win products, the demographics and socio-economic characteristics of Pay-to-Win gamers and those who purchase Pay-to-Win products have scarcely been investigated. Some studies report data about loot box purchasers, e.g. Von Meduna et al. (2020), but as loot boxes may contain items of functional or non-functional character and Pay-to-Win products are not just loot boxes, this data is not precise in describing Pay-to-Win gamers. To the best of our knowledge, Lelonek-Kuleta et al. (2021) are the only ones to report the socio-demographic profile of Pay-to-Win gamers and those who pay for Pay-to-Win products. Based on a representative sample of Poles (N = 2000), the authors report differentiating factors compared to non-gamers of Pay-to-Win to be gender, age, phase of life, education, professional status, household size and income as well as the size of their hometown. Gamers are more often male, younger on average and thus also more often unmarried students and pupils, living with their parents. Gamers are furthermore often in lower income classes and live in cities with less than 500,000 inhabitants. Compared to non-payers, they are more often female, in single-households and more often in families with children between 7 and 14 years of age than non-payers. While the study comprehensively reports on Pay-to-Win gamers, the sample size is small, and geographically limited. The question arises if the results can be confirmed for other countries and larger samples.

An early study by Wood et al. (2004) investigates the structural characteristics of video gaming, pointing out many similarities to online gambling. These include predictable and looped response stimuli, hand–eye coordination, involvement of skill, aural and visual rewards for winning and the involvement of social elements. King et al. (2015) account for the increasing challenge to structurally distinguish gambling from gaming by conceptualizing gambling and gambling-like features in games across nine primary dimensions: interactivity, monetization, betting mechanics, determination of outcome, measurement of outcome, structural fidelity, context, centrality and advertising. Given the structural similarities between gambling and video gaming, it comes as no surprise that individuals who engage in excessive gaming or gambling have similar attributes regarding their mental health (Lorains et al., 2011), demographics and personality (Mishra et al., 2017; Sanders & Williams, 2019). In light of these connections, the topics of migration and co-involvement have also been addressed. Sanders and Williams (2019) examine the relationship between video gaming and gambling in a cross-sectional sample of 3924 Canadian online panelists. Although most gamblers also played video games (70.7%) and vice versa (78.5%), (over)involvement in one of the game forms does not strongly predict (over)involvement in the other. The authors however note their failure to differentiate video gaming according to the presence of real money microtransactions that are related to gambling-like elements. Because of the missing differentiation, Pay-to-Win gaming is not explicitly investigated, which leaves the open question, if these results also hold for Pay-to-Win’s relation to gambling. It is these monetization strategies that allow players to develop the same spending behaviors as in gambling (e.g. Fiedler, 2015; Fiedler et al., 2019) and that operators use to promote player commitment, which is associated with an increased risk of excessive playing and, ultimately, addictive behaviors (Tone et al., 2014).

Macey and Hamari (2018) investigate the relationships between gaming, esports spectating and gambling using data from an international online survey of 613 respondents. Their findings indicate that gaming is not associated with an increase in problematic gambling, and the authors question the fundamental similarities between problem gaming and problem gambling. In a follow-up study, Macey and Hamari (2019) investigate the relationship between gaming involvement (esports spectatorship) and embedded gambling-like elements in games (loot box purchases). They find that most esports spectators buy loot boxes, 50.34% of whom exhibit potentially problematic gambling behavior according to the Problem Gambling Severity Index (PGSI). Macey et al. (2020) find that esports betting does not directly encourage gambling behaviors. In contrast, Lelonek-Kuleta and Bartczuk (2021) report a high prevalence of Pay-to-Win gaming and purchases among esports bettors. They find that Pay-to-Win payments are predicting problem gambling among esport bettors. The question remains whether these findings are representative and to what extent they can be generalized from video gaming specifically to Pay-to-Win gaming (Macey & Hamari, 2018), from loot box purchasers to Pay-to-Win gamers (Macey & Hamari, 2019) and from esports betting to other forms of gambling (Lelonek-Kuleta & Bartczuk, 2021).

One of the few studies to focus specifically on Pay-to-Win, as opposed to video gaming in general or social gambling games, was conducted by Dreier et al. (2017), who investigate children and adolescents aged 12–18 in a German representative study about Pay-to-Win game habits. The authors do not investigate the link to gambling but instead focus on Internet Gaming Disorder (IGD). The authors find that IGD is associated with increased involvement and spending in such games.

In summary, academic research on Pay-to-Win gaming is scarce. Most often, studies do not distinguish between Pay-to-Win and related game forms, e.g. Free-to-Play, and thus focus not specifically on Pay-to-Win but on specific products such as loot boxes, or motives to buy in-game items in general. The following explorative research questions aim at addressing the identified research gaps:

1. Motives: What are gamers’ motives for purchasing Pay-to-Win products, and which relevance has the desire to advance in the game?
2. Demographics and socio-economics: Who spends on Pay-to-Win games, how do these players differ from gamblers, and who engages in both Pay-to-Win and gambling?
3. Pay-to-Win’s relationship to gambling: How does Pay-to-Win gaming affect gambling and vice versa in terms of participation, frequency, and problematic behavior?

We explored the first two research questions using descriptive analyses. The third question on the relation of Pay-to-Win to gambling is operationalized to a set of regression models. That is, we test whether gambling-related variables can predict Pay-to-Win usage and vice versa, with regards to participation, frequency and problematic behavior. To account for the multi-faceted forms of gambling and their varying degrees of association with involvement and problem gambling (Binde et al., 2017), we used separate variables for the individual forms of gambling.

## Data and Methodology

### Dataset and Indices

The dataset was gathered as part of the e-GAMES (Electronic Gam(bl)ing: Multinational Empirical Surveys) study, which is a cross-national survey that has so far been conducted in France, Italy, Switzerland, Poland, and Germany; Canada and Australia are next. The aim is to explore the overlap between gaming and gambling and enable international comparisons based on a standardized core questionnaire and large samples. The German online panel comprised 82,985 adult internet users, representative in regard to gender and age, and was conducted between July and October 2018. 46,136 (55.6%) intended to participate, but 40,136 of them were excluded upfront for participating in neither online gambling nor Pay-to-Win gaming. 6000 respondents ultimately completed the survey. 685 respondents were later excluded for speeding through the questionnaire or for answering in suspiciously systematic patterns.

An important goal for e-GAMES is to assess the prevalence of problem gambling on national and international scales. For this, the Problem Gambling Severity Index (PGSI)(Ferris & Wynne, 2001) was used as screening tool to identify problem gamblers in the surveys. The PGSI consists of 9 items with answers reported on a 4-point Likert scale (‘never’; ‘sometimes’; ‘most of the time’; ‘almost always’). Respondents are categorized as non-problem gamblers (score of 0); low-risk gamblers (1–2); moderate-risk gamblers (3–7); or problem gamblers (8 +).

In the course of our analysis, problematic gaming behaviors also play an important role. Since the widely recognized gaming disorder (WHO, 2019) relates only to video games in general and not in particular on Pay-to-Win games, we decided against using a screening tool for gaming disorder. Instead, we use an adapted and extended PGSI solely for assessing Pay-to-Win problem gaming. The items of the standard PGSI questionnaire have been modified to the context of Pay-to-Win gaming (see Appendix 1), and by including the question ‘Have you spent more time than you intended?’, the dimension of time was incorporated. To ensure comparability between the standard PGSI and the extended PGSI, we normalized the resulting score to the standard PGSI scale [0;27].

### Description of Variables

We recorded our respondents’ socio-economic characteristics using an extensive set of variables. The variable *Age* represents a survey participants’ average age in years and, additionally, separate age groups, e.g. 18–24, are used. *Male* is a dummy variable for the participants’ gender. We use four dummy variables to describe the personal relationship status, *Single*, *Married*, *Partnership* and *Other*, which includes respondents who are widowed or divorced. *Employed* indicates whether a respondent is employed, while *Outside labor force* indicates if an individual is neither working nor looking for work, such as retirees, students or homemakers. *Unemployed* signals that a person is unemployed but not outside the labor force. Six dummy variables indicate an individual’s highest level of education, *No graduation*, *Main- or Middle school*, *A-levels, Apprenticeship* and *University*. An individual’s monthly net household income is described by another set of dummy variables: less than €500, €500–€999, €1,000–€1499, €1500–€2999, €3000–€4999 and €5000 and above. We also use the ordinal variable *Income* in the regression analyses, which indicates association with a specific income class from 1 to 6.

We use eight dummy variables to indicate whether and how often an individual participated in the following forms of online gambling during the last 12 months: *Lotteries and scratch cards*, *Slot machines*, *Poker*, *(Other) Casino games*, *Betting on horseraces*, *Sports betting*, *Esports betting* and *Financial betting*, which refers to financial products with a gambling character, e.g. binary options. For each gambling form, we create a dummy variable for participation (e.g. *Part. lotteries*) and an ordinal variable to represent the frequency of participation (e.g. *Freq. lotteries*). The assigned frequencies are 365 (‘every day’), 274 (‘nearly every day’), 156 (‘multiple times a week’), 52 (‘once a week’), 36 (‘multiple times a month’), 12 (‘once a month’) and 3 (‘less than once a month’). The variable *frequency of gambling payments*, which is also used as a dependent variable in the regression analyses, refers to payments across all forms of gambling. Similarly, the variable *frequency of Pay-to-Win payments* is based on the above-described frequencies but refers to Pay-to-Win games. The variable is used as a dependent variable in the regression analyses and refers to payments in such games, not participations.

*Cumulative spending* measures the amount of money spent on online gambling over the past 12 months. For the regression analyses we divided the variable by 1000. For each form of gambling we additionally used dummy variables (*Spend. lotteries*). With regards to the amount of money spent on Pay-to-Win games, *Cumulative spending (Pay-to-Win)* sums up the amount spent over the past 12 months. For the regression analyses we divided the variable by 1000.

For investigating Pay-to-Win players, it is important to distinguish between those players who pay and those who do not (Lelonek-Kuleta et al., 2021). But work by Alha et al. (2018) point out the importance of the motives for purchases of functional and non-functional products, i.e. in-game items, which led us to further distinguish Pay-to-Win players among the motives for their payments. All respondents who made payments in such games during the past twelve months were asked to state which one(s) from a predefined set of choices best described their motives for the payment. For each motive, we established a dummy variable: [1] ‘to significantly increase your chances to win’ (*Increase chances*), [2] ‘to win time in the game’ (*Extend game time*), [3] ‘to keep the game going’ (*Reduce pauses*), [4] ‘to enjoy the game’ (*Enhance enjoyment*), [5] ‘for aesthetic reasons (e.g. for better looking avatars)’ (*Aesthetics*), [6] ‘to support the community of the game’ (*Community support*), and [7] ‘other reasons’ (*Other*). The variable *Number of reasons* sums up the number of reasons stated by each respondent. The first three reasons for payments [1;3] are progress-related, and we collectively refer to them as *Pay-to-Win reasons*. All respondents who stated at least one of the *Pay-to-Win reasons* make up the subset of Pay-to-Win players. As such, we define Pay-to-Win gamers as those players of Pay-to-Win games, who spend money based on progress-related motives.

The dummy variables *No risk* [0], *Low risk* [1;2], *Moderate risk* [3;7] and *High risk* [8 +] indicate a respondent’s association with a specific PGSI category, based on their scores for the PGSI and the adapted PGSI for Pay-to-Win gaming. In the multivariate section, we use the actual *PGSI scores* [0;27] as dependent variables. The distribution of the PGSI score for gambling and for Pay-to-Win gaming is provided in the appendix (Appendix 2).

## Results

### Descriptive Results

#### Motives for Pay-to-Win Payments

Table 1 shows statistics on the respondent’s motives to make payments in Pay-to-Win games. Of the full sample of respondents, 55.4% (3322) have played Pay-to-Win games and of this subset of players, 1798 (54.1%) made at least one payment in such games during the past year. 1508 players of these made payments for the cause of advancing in the game (*Pay-to-Win reasons*; [1], [2], [3]). Of these, 775 stated only *Pay-to-Win reasons*, while 733 stated mixed reasons. When asked to state their primary motivation, 61.8% of the respondents with mixed reasons in the multiple-selection question selected one of the three Pay-to-Win reasons. Even for those Pay-to-Win players who make payments for a variety of reasons, the most important motivations are related to advancing in the game, especially for increasing the chances of winning. In total, 81.5% of the respondents who made at least one payment did so primarily for progress-related reasons.

**Table 1:** Reasons for payments in Pay-to-Win games

‘In the last twelve months, why have you made a payment [in Pay-to-Win games]?’	Multiple selection					Single selection	
	Multiple selection	Single selection	Pay-to-Win reasons only	Mixed reasons	Non-Pay-to-Win reasons only	Pay-to-Win reasons only	Mixed reasons
Respondents	1798	1508	775	733	290	775	733
Answers	3396	1508	995	2049	352	775	733
*Motivations*							
[1] Increase chances	22.6%	32.0%	38.3%	18.9%	0.0%	40.0%	23.6%
[2] Extend game time	21.8%	27.9%	35.3%	19.0%	0.0%	34.5%	20.9%
[3] Reduce pauses	17.2%	21.6%	26.4%	15.7%	0.0%	25.5%	17.3%
[4] Enhance enjoyment	23.3%	13.4%	0.0%	27.1%	67.0%	0.0%	27.6%
[5] Aesthetics	9.0%	3.6%	0.0%	11.7%	18.8%	0.0%	7.5%
[6] Community support	3.6%	0.9%	0.0%	5.1%	4.8%	0.0%	1.8%
[7] Other	2.5%	0.7%	0.0%	2.5%	9.4%	0.0%	1.4%

#### Socio-Economic Characteristics of Pay-to-Win Players and Gamblers

As shown in Table 2, the average age of the 1508 Pay-to-Win players is roughly 43 years, and the majority of them are female (51.1%). Most Pay-to-Win players are rather settled in relationships (64%) and are employed (73.5%). In terms of their educational attainment, 26.5% completed an apprenticeship and 24.8% passed main or middle school. The largest groups in terms of monthly net household income are €2000–2999 (21.6%) and €3000–3999 (21.3%). The prevalence of online gambling among Pay-to-Win-players is 46.4%.

**Table 2:** Descriptive results on demographics and socio-economics of Pay-to-Win gamers and gamblers

	(1)	(2)	(3)		(4)	(5)	
Sample	P2W Population (P2WP) n = 1508	Everyday P2W Buyer n = 124	Occasional P2W Buyer n = 1384	Δ t-test (2) and (3)	Online Gambler (subset of P2WP) n = 700	Online Gambler (full sample w/o P2WP) n = 4492	Δ t-test (4) and (5)
*Age*							
Average	43.09	41.02	43.36	2.34*	36.60	44.42	7.82***
18–24	10.5%	12.1%	10.4%	1.7%	14.1%	8.5%	5.6%**
25–34	23.5%	37.1%	22.3%	14.8%***	36.7%	20.5%	16.2%**
35–49	32.0%	26.6%	32.5%	5.9%	32.4%	31.9%	0.5%
50 +	34.0%	24.2%	34.8%	10.6%*	16.7%	39.1%	22.4%***
Gender (% male)	48.9%	62.1%	47.7%	14.4%**	54.7%	54.3%	0.4%***
*Personal status*							
Single	26.1%	26.6%	26.1%	0.5%	31.1%	27.1%	4.0%
Married	40.3%	43.5%	40.0%	3.6%	40.1%	40.3%	0.1%
In partnership	23.7%	17.7%	24.2%	6.5%	23.3%	21.4%	1.9%*
Other	9.9%	12.1%	9.8%	2.3%	5.4%	11.2%	5.8%
*Employment status*							
Employed	73.5%	79.7%	74.1%	5.6%	86.2%	73.3%	12.9%
Unemployed	5.6%	4.9%	5.7%	0.8%	4.0%	4.7%	− 0.6%
Outside labor force	19.5%	15.4%	20.2%	4.7%	9.8%	20.9%	11.1%
*Level of education*							
University	21.9%	17.7%	22.3%	4.5%	22.4%	26.4%	4.0%***
Apprenticeship	26.5%	20.2%	27.0%	6.9%	24.9%	31.7%	6.8%***
A-levels	21.8%	12.9%	22.5%	9.6%*	22.6%	19.8%	2.8%
Main- or middle school	24.8%	24.2%	24.9%	0.7%	24.3%	21.5%	2.8%***
No graduation	5.1%	25.0%	3.3%	21.7%***	5.9%	0.6%	5.2%***
*Net household income*							
> €5000	6.1%	8.9%	5.9%	3.0%	6.3%	7.5%	1.2%*
€3000–4999	21.4%	12.1%	22.2%	10.1%**	25.1%	25.5%	0.3%***
€2000–2999	21.6%	17.7%	21.9%	4.2%	20.7%	25.7%	5.0%***
€1500–1999	17.8%	30.6%	16.7%	14.0%***	18.3%	13.2%	5.1%***
€1000–1499	16.6%	13.7%	16.8%	3.1%	14.6%	12.2%	2.3%***
€500–999	7.8%	6.5%	7.9%	1.5%	6.9%	6.3%	0.6%**
< €500	4.7%	8.1%	4.4%	3.7%	4.4%	2.7%	1.7%***
n/a	4.0%	2.4%	4.2%	1.8%	3.7%	6.9%	3.2%***

*, **, *** indicates significance at 0.1, 0.05 and 0.01

Differentiating Pay-to-Win players according to their purchasing frequency reveals considerable heterogeneity. Relative to occasional buyers of Pay-to-Win items, daily buyers are much more likely to be male (Δ = 14.4%***) and in the young age group of 25–34-year-olds (Δ = 14.8%***). The heterogeneity is further reflected in the fact that 25% of the daily buyers have not completed any further education (Δ = 21.7%***) and are overrepresented in the lower income brackets. The share of gamblers is significantly higher among the daily buyers (Δ = 13.6%***) compared to occasional buyers.

To better understand the relationship between Pay-to-Win and online gambling, we compare online gamblers who are also Pay-to-Win players (*Pay-to-Win gamblers*; n = 700) to online gamblers who are not Pay-to-Win players (*Gamblers*, n = 4492). *Pay-to-Win gamblers* are significantly younger on average than *Gamblers* (Δ = 7.82***); accordingly, we see significant differences in the age structure, e.g. a much higher share of *Gamblers* aged 50 + (Δ = 22.4%***). 86.2% of *Pay-to-Win gamblers* are employed, compared to 73.3% of the *Gamblers*. While 5.9% of *Pay-to-Win gamblers* have not completed any further education, the corresponding share among *Gamblers* is only 0.6% (Δ = 5.2%***). Participation in Pay-to-Win games in addition to gambling further reveals lower educational levels and a lower net household income compared to *Gamblers*.

#### Prevalence, Usage and Risk Profiles of Pay-to-Win Players and Gamblers

With regards to usage profiles for Pay-to-Win gamers, there is a clear connection of the frequency of payments in Pay-to-Win games and literally every aspect of gambling. Pay-to-Win gamers who purchase daily are significantly more often participating in each of the differentiated forms of gambling, report higher frequencies of participations and higher spending than occasional buyers (Table 3). With an average PGSI score of 17.8, the daily buyers’ distribution among the PGSI groups is heavily skewed towards the high-risk category: 90.4% of the daily buyers are at high risk of developing problematic gambling behavior (Δ = 38.6%***).

**Table 3:** Descriptive results on Pay-to-Win gaming, gambling, problem gaming and gambling

	(1)	(2)	(3)		(4)	(5)	
Sample	P2W Population (P2WP) n = 1508	Everyday P2W Buyer n = 124	Occasional P2W Buyer n = 1384	Δ t-test (2) and (3)	Online Gambler (subset of P2WP) n = 700	Online Gambler (full sample w/o P2WP) n = 4492	Δ t-test(4) and (5)
*Pay-to-Win*							
Participation (payments)	100.0%	100.0%	100.0%	–	100.0%	0.0%	–
Freq. payment	95.65	365.00	71.09	293.91***	109.33	–	–
Cumulative spending	647.05	2,422.28	488.00	1,934.28***	814.90	–	–
*Problem gaming (adapted PGSI)*							
PGSI score [0;27]	8.81	14.78	8.28	6.50***	9.56	–	–
No risk	17.8%	7.3%	18.7%	11.5%***	16.0%	–	–
Low risk	11.5%	5.6%	12.1%	6.4%*	10.3%	–	–
Moderate risk	20.0%	8.1%	21.0%	13.0%***	15.9%	–	–
High risk	50.7%	79.0%	48.2%	30.8%***	57.9%	–	–
*Gambling*							
Participation	46.4%	58.9%	45.3%	13.6%***	100.0%	100.0%	–
Part. lotteries	37.2%	50.8%	36.0%	14.8%***	80.1%	83.0%	2.8%***
Part. slots	19.7%	30.6%	18.7%	11.9%***	42.4%	18.8%	23.6%
Part. poker	18.2%	33.9%	16.8%	17.1%***	39.1%	17.3%	21.9%
Part. casino	13.5%	29.8%	12.1%	17.8%***	29.1%	13.9%	15.3%
Part. horserace bet	10.3%	27.4%	8.7%	18.7%***	22.1%	6.2%	16.0%***
Part. sports bet	18.5%	30.6%	17.4%	13.2%***	39.9%	29.3%	10.5%***
Part. esports bet	11.3%	25.8%	10.0%	15.8%***	24.3%	7.1%	17.1%***
Part. financial bet	7.8%	20.2%	6.7%	13.4%***	16.9%	7.2%	9.6%
Freq. payment	181.08	725.06	132.34	592.73***	390.09	116.85	273.23***
Freq. lotteries	47.60	153.26	38.14	115.12***	102.55	43.08	59.48**
Freq. slots	29.67	100.60	23.31	77.29***	63.92	16.05	47.87***
Freq. poker	26.32	108.17	18.98	89.18***	56.70	13.90	42.80***
Freq. casino	18.83	84.51	12.95	71.56***	40.57	9.43	31.14***
Freq. horserace bet	13.24	69.73	8.18	61.56***	28.52	3.70	24.82***
Freq. sports bet	19.72	74.47	14.82	59.65***	42.48	19.79	22.70
Freq. esports bet	15.69	74.01	10.46	63.55***	33.79	4.54	29.25***
Freq. financial bet	10.01	60.31	5.50	54.82***	21.56	6.38	15.18**
Cumulative spending	331.16	1,273.01	246.77	1,026.23***	713.41	203.82	509.59***
Spend. lotteries	76.36	279.02	58.20	220.81***	164.50	58.10	106.39**
Spend. slots	48.90	151.23	39.73	111.49***	105.34	36.25	69.10
Spend. poker	53.68	231.40	37.76	193.65***	115.64	30.82	84.82***
Spend. casino	24.91	86.53	19.39	67.14***	53.67	13.18	40.49***
Spend. horserace bet	34.85	213.41	18.85	194.56***	75.08	6.67	68.41***
Spend. sports bet	43.08	108.56	37.21	71.35**	92.80	30.69	62.11*
Spend. esports bet	20.91	74.80	16.08	58.72***	45.04	6.39	38.66***
Spend. financial bet	28.47	128.06	19.55	108.51***	61.34	21.73	39.61
*Problem gambling (PGSI)*							
PGSI score [0;27]	9.45	17.77	8.48	9.29***	9.45	3.13	6.32***
No risk	11.1%	0.0%	12.4%	12.4%***	11.1%	40.6%	29.5%***
Low risk	11.7%	2.7%	12.8%	10.0%**	11.7%	25.9%	14.2%***
Moderate risk	21.3%	6.8%	23.0%	16.1%***	21.3%	17.6%	3.7%**
High risk	55.9%	90.4%	51.8%	38.6%***	55.9%	15.9%	40.0%***

*, **, *** indicates significance at 0.1, 0.05 and 0.01

The group comparison of gamblers who also play Pay-to-Win games and gambler who do not reveals that the former group is participating significantly more often in less-prominent forms of gambling, e.g. horserace betting and esports betting. Furthermore, the frequency of payment is significantly higher across all forms of gambling, except sports betting, for *Pay-to-Win gamblers*. With regards to spending, financial betting and slots are the only gambling forms for which significantly higher spending of *Pay-to-Win gamblers* have not been found. In line with these results, 55.9% of *Pay-to-Win gamblers* are classified as high-risk gamblers according to the PGSI, while the same applies to only 15.9% of the *Gamblers* (Δ = 40%***).

### Multivariate Analysis Results

#### Predicting Participation in Pay-to-Win and Gambling

Participation in some forms of gambling–specifically online slots, poker, online casino games, horserace and esports betting–is associated with significant increases in the odds ratios for participating in Pay-to-Win games, while the results for lotteries, sports betting and financial betting are not significant (Table 4). This could be explained by the structural differences between the various forms of gambling, e.g. betting versus slots, poker or casino games. The latter gambling forms can be regarded more similar to those mechanics which can be found in Pay-to-Win games, and they have a positive and significant impact on Pay-to-Win participation. Similarly, the influence of the frequency of payments differs across the forms of gambling.

**Table 4:** Regressions predicting participation in Pay-to-Win games and gambling

Model (Binary logistic regression)	1		2		3		4	
Dependent variable	Pay-to-Win participation				Gambling participation			
Sample	Total sample				Pay-to-Win gamers			
	n = 4852		n = 4731		n = 1426		n = 1426	
	Coeff.(SE)	Std. Coeff	Coeff.(SE)	Std. Coeff	Coeff.(SE)	Std. Coeff	Coeff.(SE)	Std. Coeff
Age	− 0.023(0.004)***	− 0.320	− 0.025(0.004)***	− 0.348	− 0.066(0.005)***	− 0.992	− 0.068(0.005)***	− 1.020
Male	− 0.239(0.097)**	− 0.119	− 0.202(0.098)**	− 0.101	0.446(0.125)***	0.223	0.451(0.125)***	0.226
*Personal status*								
Single	–	–	–	–	–	–	–	–
Married	0.166(0.127)	0.082	0.119(0.130)	0.058	0.213(0.172)	0.105	0.233(0.174)	0.115
Partnership	0.151(0.130)	0.062	0.117(0.133)	0.048	− 0.239(0.178)	− 0.101	− 0.232(0.179)	− 0.098
Other	− 0.211(0.213)	− 0.065	− 0.239(0.215)	− 0.074	− 0.274(0.262)	− 0.083	− 0.266(0.264)	− 0.081
*Employment status*								
Employed	–	–	–	–	–	–	–	–
Unemployed	− 0.822(0.239)***	− 0.173	− 0.703(0.242)***	− 0.147	− 0.546(0.286)**	− 0.127	− 0.504(0.290)**	− 0.117
Outside labor force	− 0.671(0.149)***	− 0.269	− 0.642(0.151)***	− 0.257	− 0.816(0.180)***	− 0.330	− 0.794(0.181)***	− 0.321
*Education*								
University	− 1.820(0.317)***	− 0.798	− 1.236(0.350)***	− 0.542	− 0.810(0.341)**	− 0.336	− 0.724(0.338)**	− 0.300
Apprenticeship	− 1.738(0.312)***	− 0.805	− 1.201(0.344)***	− 0.558	− 0.290(0.332)	− 0.128	− 0.208(0.330)	− 0.092
A-levels	− 1.812(0.313)***	− 0.723	− 1.285(0.346)***	− 0.511	− 0.644(0.340)*	− 0.263	− 0.573(0.338)*	− 0.234
Main- or middle school	− 1.380(0.310)***	− 0.569	− 0.825(0.344)**	− 0.339	− 0.109(0.333)	− 0.047	− 0.053(0.331)	− 0.023
No graduation	–	–	–	–	–	–	–	–
Net household income	− 0.087(0.037)**	− 0.132	− 0.085(0.038)**	− 0.128	0.120(0.047)***	0.190	0.122(0.047)***	0.192
*Reasons for P2W payments*								
Number of reasons					− 0.082(0.058)	− 0.089		
Increase chances							–	–
Extend game time							− 0.225(0.200)	− 0.077
Reduce pauses							− 0.597(0.226)***	− 0.181
Enhance enjoyment							− 0.153(0.324)	− 0.031
Aesthetics							− 0.949(0.494)**	− 0.123
Community support							− 1.493(0.763)**	− 0.122
Other							− 0.694(0.970)	− 0.047
*Pay-to-Win*								
Freq. payments					0.000(0.001)	0.030	0.000(0.001)	0.015
Cumulative spending					0.036(0.031)	0.076	0.036(0.030)	0.077
*Gambling*								
Part. lotteries	0.115(0.117)	0.043						
Part. slots	0.298(0.108)***	0.124						
Part. poker	0.321(0.110)***	0.130						
Part. casino	0.313(0.117)***	0.115						
Part. horserace bet	0.470(0.152)***	0.131						
Part. sports bet	− 0.059(0.107)	− 0.027						
Part. esports bet	0.250(0.143)*	0.073						
Part. financial bet	0.009(0.158)	0.003						
Freq. lotteries			0.003(0.001)***	0.247				
Freq. slots			0.002(0.001)**	0.104				
Freq. poker			0.002(0.001)***	0.135				
Freq. casino			0.002(0.001)***	0.121				
Freq. horserace bet			0.001(0.001)	0.040				
Freq. sports bet			− 0.001(0.001)*	− 0.083				
Freq. esports bet			0.002(0.001)**	0.093				
Freq. financial bet			− 0.001(0.001)	− 0.032				
Cumulative spending	0.059(0.040)	0.053						
*Problem gam(bl)ing*								
High risk (P2W)					0.302(0.131)**	0.151	0.307(0.131)**	0.153
High risk (gambling)	1.267(0.103)***	0.523	1.159(0.107)***	0.477				
Nagelkerke R^2^	0.239	0.257	0.299	0.307				

'-' indicates omitted variables; *, **, *** indicates significance at 0.1, 0.05 and 0.01; Constant included but not reported

Engaging regularly in lotteries, slots, poker, online casino games and esports betting is positively associated with Pay-to-Win participation. Also with regards to frequencies, the results suggest that some forms of gambling are structurally closer to Pay-to-Win games than others, e.g. sports betting, horserace betting and financial betting. Cumulative spending on online gambling does not have a significant impact. By contrast, being a high-risk gambler according to the PGSI significantly increases the probability of playing Pay-to-Win games.

In both of the models 3 and 4, neither the frequency of payments nor the cumulative amount spent in Pay-to-Win games has a significant impact on gambling participation. Regarding the reasons for payments in Pay-to-Win games, none of them exhibit a significant positive impact. By contrast, individuals who are at risk of problematic gaming behavior are significantly more likely to gamble. In sum, Pay-to-Win gaming does not significantly predict gambling participation in general. On the behavioral level, however, problematic usage patterns may lead excessive Pay-to-Win players to gamble and vice versa.

#### Predicting the Frequency of Pay-to-Win and Gambling Spending

The OLS regression models 5 through 8 allow us to examine the influence of gambling-related variables on the frequency of payments in Pay-to-Win games and the influence of Pay-to-Win on the frequency of gambling payments. Models 5 and 6 determine the predictors of the frequency of making payments in Pay-to-Win games (Table 5). In both models, participation in lotteries and the frequency of playing lotteries are the strongest influences on the frequency of payments in Pay-to-Win games, relative to other forms of gambling. While participation in none of the other gambling forms, except for poker, has a significant influence, the frequency of participation does matter significantly for slots, poker and online casino games. In model 5, the cumulative spending on gambling has a strong positive influence on the Pay-to-Win payment frequency. Being in the PGSI high-risk group for problematic gambling behavior is also associated with a high frequency of payments in Pay-to-Win games.

**Table 5:** Regression models predicting the frequency of Pay-to-Win and gambling payments

Model (OLS)	5		6		7		8	
Dependent variable	Frequency of Pay-to-Win payments				Frequency of gambling payments			
Sample	Gambler and Pay-to-Win gamers							
	n = 665		n = 650		n = 1425		n = 1425	
	Coeff.(SE)	Std. Coeff	Coeff.(SE)	Std. Coeff	Coeff.(SE)	Std. Coeff	Coeff.(SE)	Std. Coeff
Age	− 0.779(0.358)**	− 0.076	− 0.571(0.320)*	− 0.056	− 6.224(0.725)***	− 0.223	− 6.195(0.740)***	− 0.222
Male	2.800(7.788)	0.011	− 4.359(7.032)	− 0.018	80.144(18.801)***	0.096	78.145(19.164)***	0.093
*Personal status*								
Single	–	–	–	–	–	–	–	–
Married	12.542(10.108)	0.051	5.453(9.222)	0.022	94.457(26.425)***	0.111	100.094(26.889)***	0.118
Partnership	− 7.184(10.640)	− 0.025	− 6.079(9.733)	− 0.021	− 17.051(27.485)	− 0.017	− 16.784(28.027)	− 0.017
Other	46.147(17.693)**	0.087	40.975(15.939)***	0.078	41.156(36.714)	0.030	29.873(37.376)	0.022
*Employment status*								
Employed	–	–	–	–	–	–	–	–
Unemployed	− 27.631(19.639)	− 0.046	− 15.275(17.906)	− 0.025	− 84.782(42.440)**	− 0.047	− 77.734(43.456)*	− 0.043
Outside labor force	− 10.111(13.010)	− 0.025	− 8.164(11.827)	− 0.020	− 69.508(25.587)***	− 0.067	− 70.049(26.131)***	− 0.068
*Education*								
University	− 128.915(17.579)***	− 0.440	− 73.718(16.917)***	− 0.251	− 237.207(47.654)***	− 0.235	− 281.447(48.158)***	− 0.278
Apprenticeship	− 145.362(17.284)***	− 0.521	− 88.435(16.694)***	− 0.317	− 227.641(46.054)***	− 0.240	− 263.814(46.699)***	− 0.278
A-levels	− 158.069(17.368)***	− 0.533	− 104.392(16.949)***	− 0.349	-228.611(47.143)***	− 0.223	− 265.260(47.839)***	− 0.259
Main- or middle school	− 129.079(17.526)***	− 0.547	− 72.503(17.008)***	− 0.255	− 237.123(45.974)***	− 0.245	− 283.092(46.395)***	− 0.293
No graduation	–	–	–	–	–	–	–	–
Net household income	0.153(3.000)	0.002	0.908(2.724)	0.012	3.646(7.015)	0.014	6.097(7.144)	0.023
*Reasons for P2W payments*								
Number of reasons					66.167(8.709)***	0.172		
Increase chances							–	–
Extend game time							− 23.152(30.827)	− 0.019
Reduce pauses							0.162(34.359)	0.000
Enhance enjoyment							87.778(50.081)*	0.042
Aesthetics							144.564(74.072)*	0.045
Community support							253.025(115.092)**	0.049
Other							− 106.923(139.579)	− 0.017
*Pay-to-Win*								
Freq. payments					1.100(0.090)***	0.302	1.146(0.092)***	0.315
Cumulative spending					9.511(4.561)**	0.048	9.508(4.642)**	0.048
Gambling								
Part. lotteries	23.662(9.436)**	0.078						
Part. slots	− 4.416(8.461)	− 0.018						
Part. poker	14.666(8.831)*	0.059						
Part. casino	6.954(9.096)	0.026						
Part. horserace bet	15.652(11.723)	0.054						
Part. sports bet	− 5.512(8.585)	− 0.022						
Part. esports bet	− 13.458(10.754)	− 0.047						
Part. financial bet	− 8.954(12.297)	− 0.027						
Freq. lotteries			0.260(0.035)***	0.260				
Freq. slots			0.084(0.040)**	0.076				
Freq. poker			0.191(0.043)***	0.263				
Freq. casino			0.140(0.046)***	0.107				
Freq. horserace bet			− 0.057(0.067)	− 0.038				
Freq. sports bet			0.052(0.049)	0.038				
Freq. esports bet			0.015(0.057)	0.011				
Freq. financial bet			0.022(0.066)	0.012				
Cumulative spending	15.353(2.816)***	0.192						
*Problem gam(bl)ing*								
High risk (P2W)					66.832(19.745)***	0.080	76.076(20.053)***	0.091
High risk (gambling)	96.29(8.511)***	0.393	66.912(7.817)***	0.272				
R^2^(adj)	0.417(0.397)	0.531(0.516)	0.334(0.326)	0.314(0.303)				

'-' indicates omitted variables; *, **, *** indicates significance at 0.1, 0.05 and 0.01; Constant included but not reported

Models 7 and 8 predict the frequency of gambling payments. In both models, the frequency of payments in Pay-to-Win games has a significant influence on the frequency of payments in gambling. Cumulative spending in Pay-to-Win games likewise predicts the frequency of gambling payments, and excessive gaming behavior also has a strong influence. Significantly more gambling payments are made by those Pay-to-Win players who report multiple reasons for making payments in games: An additional reason for payments raises the frequency of gambling payments by 66 times per year. As for the explicit purchase motivations, increasing the chances of winning or advancing in the game was excluded to serve as the reference group. Surprisingly, for the remaining motivations, we find significant positive influences for non-Pay-to-Win-related reasons, most importantly for *Community support*.

#### Predicting Problematic Pay-to-Win Gaming and Gambling Behavior

Table 6 shows the results of models 9 through 12, which investigate the predictive power of gambling-related variables on the PGSI score [0;27] for Pay-to-Win gaming (models 9 and 10) and vice versa for gambling (models 11 and 12).

**Table 6:** OLS regressions predicting the (adapted) PGSI score for Pay-to-Win gaming and for gambling

Model (OLS)	9		10		11		12	
Dependent variable	PGSI P2W Score [0;27]				PGSI Gambling Score [0;27]			
Sample	Gamblers and Pay-to-Win gamers							
	n = 672		n = 657		n = 665		n = 665	
	Coeff.(SE)	Std. Coeff	Coeff.(SE)	Std. Coeff	Coeff.(SE)	Std. Coeff	Coeff.(SE)	Std. Coeff
Age	− 0.006(0.017)	− 0.009	− 0.001(0.017)	− 0.002	− 0.039(0.017)**	− 0.063	− 0.037(0.017)**	− 0.060
Male	0.227(0.379)	0.015	0.101(0.372)	0.007	0.970(0.359)***	0.065	0.938(0.360)***	0.063
*Personal status*								
Single	–	–	–	–	–	–	–	–
Married	0.670(0.492)	0.045	0.527(0.487)	0.036	2.096(0.472)***	0.140	2.063(0.474)***	0.138
Partnership	− 0.259(0.517)	− 0.015	− 0.101(0.514)	− 0.006	0.719(0.502)	0.041	0.661(0.505)	0.038
Other	2.879(0.866)***	0.090	2.633(0.847)***	0.083	1.395(0.837)*	0.043	1.369(0.838)	0.042
*Employment status*								
Employed	–	–	–	–	–	–	–	–
Unemployed	0.205(0.962)	0.006	0.655(0.952)	0.018	− 0.449(0.934)	− 0.012	− 0.487(0.936)	− 0.013
Outside labor force	− 0.810(0.631)	− 0.034	− 0.573(0.622)	− 0.024	0.094(0.615)	0.004	0.150(0.617)	0.006
*Education*								
University	− 4.239(0.860)***	− 0.242	− 3.284(0.899)***	− 0.188	− 1.375(0.875)	− − 0.077	− 1.421(0.864)*	− 0.080
Apprenticeship	− 4.979(0.847)***	− 0.297	− 4.082(0.888)***	− 0.245	− 1.906(0.865)**	− 0.113	− 1.961(0.858)**	− 0.116
A-levels	− 4.747(0.850)***	− 0.269	− 3.858(0.900)***	− 0.217	− 1.379(0.880)	− 0.077	− 1.427(0.874)	− 0.079
Main- or middle school	− 3.436(0.858)***	− 0.203	− 2.326(0.904)***	− 0.137	− 1.053(0.876)	− 0.061	− 1.141(0.863)	− 0.067
No graduation	–	–	–	–	–	–	–	–
Net household income	0.059(0.146)	0.013	0.032(0.144)	0.007	− 0.325(0.140)**	− 0.069	− 0.328(0.142)**	− 0.069
*Reasons for P2W payments*								
Number of reasons					0.166(0.158)	0.027		
Increase chances							−	−
Extend game time							0.170(567)	0.008
Reduce pauses							0.685(0.673)	0.027
Enhance enjoyment							− 0.012(0.938)	0.000
Aesthetics							2.045(1.462)	0.035
Community support							2.558(2.344)	0.026
Other							− 3.968(2.794)	− 0.034
*Pay-to-Win*								
Freq. payments					0.018(0.002)***	0.296	0.018(0.002)***	0.298
Cumulative spending					− 0.219(0.074)***	− 0.074	− 0.218(0.074)***	− 0.074
*Gambling*								
Part. lotteries	0.187(0.461)	0.010						
Part. slots	− 0.202(0.414)	− 0.014						
Part. poker	0.447(0.432)	0.030						
Part. casino	0.156(0.446)	0.010						
Part. horserace bet	0.908(0.574)	0.052						
Part. sports bet	− 0.249(0.420)	− 0.017						
Part. esports bet	− 0.773(0.527)	− 0.045						
Part. financial bet	0.949(0.602)	0.049						
Freq. lotteries			0.006(0.002)***	0.108				
Freq. slots			0.001(0.002)	0.011				
Freq. poker			0.006(0.002)***	0.084				
Freq. casino			− 0.001(0.002)	− 0.012				
Freq. horserace bet			0.003(0.004)	0.036				
Freq. sports bet			0.002(0.003)	0.029				
Freq. esports bet			− 0.006(0.003)**	− 0.077				
Freq. financial bet			0.007(0.004)**	0.069				
Cumulative spending	0.403(0.138)***	0.084						
*Problem gam(bl)ing*								
High risk (P2W)					8.438(0.413)***	0.564	8.403(0.415)***	0.562
High risk (gambling)	9.972(0.414)***	0.681	9.556(0.413)***	0.651				
R2(adj)	0.608(0.595)	0.628(0.615)	0.641(0.632)	0.644(0.632)				

'-' indicates omitted variables; *, **, *** indicates significance at 0.1, 0.05 and 0.01; Constant included but not reported

We find that participation in a specific form of gambling is not associated with a higher individual PGSI score and thus the probability of becoming a problematic Pay-to-Win gamer. Regarding the frequencies of the gambling forms, four out of eight have significant impacts, but all of them are negligible in effect size. For example, increasing the frequency of playing lotteries by one level, e.g. from ‘multiple times per week’ to ‘nearly every day’, raises the PGSI score for Pay-to-Win gaming by 0.006 points. For comparison, increasing cumulative gambling spending by €1000 per year raises the PGSI score by 0.4 points. Unambiguously, problematic gambling behavior significantly increases the risk of problematic behavior in gaming as well.

In models 11 and 12, we similarly find mixed results in the attempt to predict the risk of problematic gambling behavior through Pay-to-Win-related variables. While problematic gaming certainly influences problematic gambling behavior in both models, the motivations to make payments in Pay-to-Win games play no important role. The influence of the frequency of payments in Pay-to-Win games is noticeable and highly significant. Increasing the frequency by ten days per year increases the risk of problematic gambling behavior by 0.16 points on the PGSI score. Surprisingly, cumulative spending in Pay-to-Win games has a significant negative effect on the PGSI score in both models 11 and 12.

## Discussion

Because of the scarce literature on Pay-to-Win gaming, our analyses are of explorative character. In consequence, our results must predominantly be discussed with reference to the existing literature, e.g. on loot boxes or in-game items in general, and gaming or gambling in general.

The identification of Pay-to-Win gamers was based on the respondents’ selection of motives which led them to pay for Pay-to-Win products. As such, we were able to reduce the sample of interest to Pay-to-Win players, who conduct payments in the game and for progress-related reasons. Referring to the first research question, we found that the desire to advance in the game is the major motive to make in-game purchases in Pay-to-Win games. Our method and finding extend the work of Hamari and Keronen (2016) and Hamari et al. (2017) on the general motivations to pay for in-game items to the context of Pay-to-Win. We also provide quantitative confirmation of the qualitative work by Alha et al. (2018) on the motivational differences concerning functional and non-functional items.

With reference to the second research question, the descriptive analyses show a distinct demographic and socio-economic profile of Pay-to-Win users in Germany that are similar to the findings for Polish gamers provided by Lelonek-Kuleta et al. (2021). Little more than half of the Pay-to-Win gamers in Germany who make purchases are female, aged in their early forties on average and employed. Differences were found in German players being lesser educated, having higher incomes and being single more often than Polish players. These deviations, however, are marginal and might be attributable to regional differences or different sample sizes. In comparison to other types of gaming, Pay-to-Win gamers are also different from profiles of, for example, loot box purchasers. Pay-to-Win gamers are older on average, more often married and less often employed than loot box users in Germany (Von Meduna et al., 2020).

Further exploring the second research question, we were separating samples according to their frequency of Pay-to-Win payments and according to their participation in gambling. Compared to occasional buyers, heavy users, i.e. daily buyers, of Pay-to-Win products are spending significant amounts of money on such games. Based on our adapted PGSI for Pay-to-Win gaming, we identify a great share that faces a high risk for developing problematic behavioral gaming patterns. This implies that Pay-to-Win games exhibit risks for over-involvement.

Pay-to-Win gamers differ to gamblers in that they are younger, more often female, more often unemployed and have lower educational levels and income. While Mishra et al. (2017) and Sanders and Williams (2019) found similar demographics of gamers and gamblers in general, the comparison of our samples of Pay-to-Win players to online gamblers who do not engage in such games suggests that this finding does not hold in the context of Pay-to-Win. More substantial differences were found in comparing the samples of Pay-to-Win players who also gamble and sole gamblers. Those players participating in both forms are significantly younger on average, are lesser educated and have lower incomes than sole gamblers. Many socio-economic differences, however, could be attributable to their younger age. On the other hand, players of both forms show a considerable intensity when gambling: the participation and frequencies of playing as well as spending differ significantly across almost all forms of gambling compared to sole gamblers. In contrast to findings by Macey et al. (2020) that esports betting does not directly encourage gambling behaviors, our results suggest that this might not hold for Pay-to-Win games. As more than half of the players of both are associated with a high-risk group for problem gambling and problem gaming, the conclusion by Macey and Hamari (2018) about a lack of fundamental similarities between problem gambling and problem gaming are, at least, questionable in the context of Pay-to-Win gaming.

Although almost half of the Pay-to-Win players also gamble, the mere participation in Pay-to-Win does not predict participation in gambling. Conversely, however, participation in specific forms of gambling, i.e. slots, poker and casino games, significantly increases the probability of Pay-to-Win gaming. Game forms that involve betting (on sports, horseraces, esports) seem to have no significant effects, which clearly shows that any investigation of the similarities between gaming and gambling should consider individual forms of gambling (Binde et al., 2017). Most importantly, we identify the frequency of payments in both Pay-to-Win games and gambling (lotteries, slots, poker and casino games) to be a strong predictor of high frequencies in the other game form. As such, and in line with the above-described results, the unlimited frequency of payments in Pay-to-Win games is identified to be an important link to gambling and thus bears risk for gamers. This is in line with statements by King et al. (2019) which point out that the frequency of microtransactions in other game forms can induce excessive involvement. This is an important addition to the vast literature that found no trajectory effect between gaming and gambling.

Our results show that the mere involvement in one game form is not a predictor for (over)involvement in the other (Sanders & Williams, 2019). But when it comes to over-involvement, our findings show that for Pay-to-Win gaming and gambling, (over)involvement in one form certainly predicts (over)involvement in the other. For Pay-to-Win players and gamblers alike, developing problematic behaviors significantly increases the chances of developing these for the other game form. Across all our analyses, the association with high-risk PGSI-categories is a strong predictor for respondents to increase participation, frequencies and PGSI-scores in the other form. Also in relative measures, the standardized coefficients disclose that the associations with high-risk PGSI-groups present the most influential variables within the different models. This can be interpreted that once a player or gambler has developed a problematic behavioral pattern, that habit is likely to migrate to other game forms. By virtue of our differentiation between Pay-to-Win gaming and general video gaming, we are thus able to refine Macey and Hamari’s (2018) finding that gaming is not associated with an increased potential for problem gambling. Instead, by finding a clear link between Pay-to-Win purchases and an increased risk of problem gambling, we confirm the findings by Leloek-Kuleta et al. (2021) for the German population.

Counterintuitively, the frequency of payments both in gambling and Pay-to-Win games is more influential than cumulative spending and general participation when it comes to predicting the participation and the playing frequency of the other game type. This suggests that absolute spending does not reflect the relative importance of the amount spent to an individual user. A more meaningful indicator might be spending in relation to an individual’s income or wealth.

The discussion reveals that Pay-to-Win gaming yields considerable relations to specific forms of gambling and the role of over-involvement. Through our approach of differentiating Pay-to-Win as a form of gaming and specific forms of gambling, our results refine the literature on the similarities of gaming and gambling and lay foundational work for analyses focusing on Pay-to-Win specifically.

## Limitations

The survey is subject to several limitations. As with any other cross-sectional study design, our study is at risk for selection bias and information bias (Wang & Cheng, 2020). Importantly, it was conducted exclusively online, providing no scope for personal instructions to and queries from the participants. Internet users who volunteer for a panel are a minority of all internet users, of whom they may not be representative. While our sample is representative of German internet users in terms of age and gender, the proportion of gamers and gamblers in our survey may be larger than the corresponding proportion across all internet users because perhaps our target group of internet users is more active and tech savvy than the average internet user. Besides such sampling bias, the study may also inherit information bias, e.g. in the form of observer bias or recall bias.

In consequence, the cross-sectional character of this study implicates limitations in the generalizability of the results and their interpretation. There are predictive limitations of cross-sectional study designs because it is not possible to establish a true relationship of cause and effect without longitudinal data (Solem, 2015), as in cross-sectional data, outcome and exposure variables are assessed simultaneously (Carlson & Morrison, 2009). As such, the statistical relations of Pay-to-Win gaming and gambling variables found in our investigation are to be interpreted with care and further research is needed to confirm our findings. The present study design is of explorative character and seeks to create a foundational investigation on the phenomenon which aims at informing and encouraging further research.

With regards to accuracy of the data, it needs to be pointed out that the questionnaire only asked about total household income but not about the number of persons in the household. Players from larger households may on the one hand have more money available for gaming or gambling; on the other hand, they will tend to have less time to gamble or play and be more risk averse.

Absent a validated tool for gaming-related problems due to Pay-to-Win usage, we have adapted the PGSI to cover gaming-related problematic behavior. The mathematical adjustment results in a more sensitive classification for cases between the values of 7 and 8. More importantly though, the adapted PGSI is not a validated screening tool. In consequence, and in line with the PGSI for gambling, the adapted PGSI can only serve as an indicator of potential problems and must be interpreted with caution.

## Conclusion and Outlook

Against the background of the ongoing convergence of gaming and gambling, Pay-to-Win presents a scarcely researched phenomenon, whose relation and similarity to gambling is still not fully understood. The objective of this paper was to shed light onto this research gap by exploring potential similarities of Pay-to-Win and gambling. For this, the phenomenon of Pay-to-Win gaming, as such, required fundamental investigations at first, as the demographic and socio-economic characteristics of users and the relevance of their motives to make payments in these games have scarcely been researched as of today.

Pay-to-Win gamers are a distinct consumer group with a clear demographic and socio-economic profile. We found important criteria for differentiations among Pay-to-Win players to be the spending of money on Pay-to-Win products, the motives for conducting payments and the frequency of spending (as opposed to the amount of money spent). These criteria allowed for identifying the especially vulnerable group of players, who are frequently spending for progress-related reasons. This procedure isolates the players of highest interest for our research and can serve as a selection procedure for future research on Pay-to-Win gaming and its relation to gambling. Against this background, future research might as well address the interrelation of different products of Pay-to-Win and purchasers’ motives. In the light of our ambiguous results regarding the relation of motives for Pay-to-Win payments and gambling, future research might address the interrelation of progress-related motives and gambling intensity.

Although Pay-to-Win gamers are different to gamblers in demographic and socio-economic characteristics, the descriptive results revealed a high prevalence of gambling among gamers. The prevalence of gambling and problem gambling increases with higher frequencies of spending in Pay-to-Win games. Based on this finding, we investigated the effects that participation, frequency of payments and problematic behavior in Pay-to-Win have on gambling and vice versa. The results of these analyses reveal several important findings: As opposed to the mere participation in Pay-to-Win gaming or gambling, the frequency of spending seems to be the most important predictor as well as the association with problematic behavior in one of the game forms. This implies that once a player has developed a problem in one game form, the probability increases that similar patterns are developed in the other form as well. Further, Pay-to-Win gaming and gambling can be substitutes for players seeking to wager or spend money frequently. Third, different effect sizes and significance for specific forms of gambling on Pay-to-Win gaming indicate that the differentiation of gambling forms is a crucial prerequisite for further research in the field.

We were thus able to show that the relation of Pay-to-Win and gambling (and the harmfulness of Pay-to-Win gaming itself) is grounded on similar game-designs which facilitate the development of problematic spending behaviors. The relation of Pay-to-Win and gambling is less obvious than the relation of, for example, loot boxes and gambling. These findings have several implications for regulators: Regulatory measures can target Pay-to-Win games to limit the frequencies of spending. Moreover, specific user groups can be targeted for educational prevention measures with a focus on spending in online games or migrations from gaming to gambling. As the most vulnerable group, i.e. players participating in Pay-to-Win gaming and gambling, is considerably young, youth protection measures, i.e. age verification for payments, must also be taken into account for discussion. In sum, our findings suggest that expanding the public discussion from specific products, e.g. loot boxes, to the broader domain of Pay-to-Win gaming is needed.

## Appendix 1

See Table 7

**Table 7:** The questionnaire for sourcing the adapted PGSI for Pay-to-Win gaming; Question: ‘Thinking about your habits only on these Pay2Win games you spent real money for during the past 12 months. To what extent do the following questions apply to you?’

	Never	Sometimes	Most of the time	Almost always
1. Have you spent more money than you could really afford to lose?				
2. Have you spent more time than you intended?				
3. Have you needed to play longer to get the same feeling of excitement?				
4. When you played and lost, did you increase your playing time to regain your initial position?				
5. Have you borrowed money or sold anything to get money to game?				
6. Have you felt that you might have a problem with gaming?				
7. Has your gaming caused you any health problems, including stress or anxiety?				
8. Have people criticized your gaming or told you that you had a gaming problem, regardless of whether or not you thought it was true?				
9. Has your gaming caused any financial problems for you or your household?				
10. Have you felt guilty about the way you game or what happens when you play?				

## Appendix 2

See Table 8

**Table 8:** Distribution of the respondents’ PGSI score for gambling and Pay-to-Win

PGSI Score (Gambling)	Frequency	Percent	Cumulative percent	PGSI Score (Pay-to-Win)	Frequency	Percent	Cumulative percent
0	1,903	31.7	36.7	0.0	186	3.1	12.3
1	788	13.1	51.8	0.9	82	1.4	17.8
2	456	7.6	60.6	1.8	83	1.4	23.3
3	316	5.3	66.7	2.7	91	1.5	29.3
4	214	3.6	70.8	3.6	48	0.8	32.5
5	159	2.7	73.9	4.5	82	1.4	37.9
6	135	2.3	76.5	5.4	62	1.0	42.0
7	116	1.9	78.7	6.3	45	0.8	45.0
8	80	1.3	80.3	7.2	64	1.1	49.3
9	215	3.6	84.4	8.1	58	1.0	53.1
10	80	1.3	86.0	9.0	105	1.8	60.1
11	80	1.3	87.5	9.9	27	0.4	61.9
12	99	1.7	89.4	10.8	39	0.7	64.5
13	101	1.7	91.4	11.7	56	0.9	68.2
14	75	1.3	92.8	12.6	67	1.1	72.6
15	78	1.3	94.3	13.5	74	1.2	77.5
16	55	0.9	95.4	14.4	35	0.6	79.8
17	39	0.7	96.1	15.3	32	0.5	82.0
18	51	0.9	97.1	16.2	26	0.4	83.7
19	25	0.4	97.6	17.1	32	0.5	85.8
20	23	0.4	98.0	18.0	40	0.7	88.5
21	19	0.3	98.4	18.9	18	0.3	89.7
22	19	0.3	98.7	19.8	18	0.3	90.8
23	31	0.5	99.3	20.7	16	0.3	91.9
24	17	0.3	99.7	21.6	15	0.3	92.9
25	3	0.1	99.7	22.5	51	0.9	96.3
26	1	0.0	99.7	23.4	18	0.3	97.5
27	13	0.2	100.0	24.3	6	0.1	97.9
				25.2	3	0.1	98.1
				26.1	10	0.2	98.7
				27.0	19	0.3	100.0
N	5,191	86.5			1,508	25.1	
Missing values	809	13.5			4,492	74.9	
Mean	3.98				8.81		
